# Automatic Suppression Reduces Anxiety-Related Overestimation of Time Perception

**DOI:** 10.3389/fphys.2020.537778

**Published:** 2020-10-22

**Authors:** Jiajin Yuan, Lingjing Li, Yu Tian

**Affiliations:** ^1^Institute of Brain and Psychological Sciences, Sichuan Normal University, Chengdu, China; ^2^Research Center for Psychology and Social Development, Southwest University, Chongqing, China

**Keywords:** time perception, anxiety, deliberate suppression, automatic suppression, attention

## Abstract

Anxiety has been found to lengthen time perception, especially the time perception of negative stimuli. This anxiety-related time overestimation is thought to be mainly associated with massively increased arousal. Suppression, which can be achieved either deliberately or automatically, has been demonstrated to be effective in reducing arousal. Consequently, the present study explored the effectiveness of both deliberate suppression (Experiment 1) and automatic suppression (Experiment 2) in reducing the time distortion in anxiety. A temporal bisection task (TBT), featuring negative and neutral pictures, was used to measure time perception, while the self-reported arousal was used to assess arousal. The deliberate suppression was manipulated by asking participants to suppress their emotional expressions; while automatic suppression was manipulated through a sentence-unscrambling task featuring suppression-related words, which can unconsciously prime suppression. The results of Experiment 1 showed that deliberate suppression did not reduce the anxiety-related time overestimation and arousal. However, Experiment 2 showed that automatic suppression significantly reduced the anxiety-related time overestimation, with significant arousal reduction being observed. In conclusion, automatic suppression, but not deliberate suppression, is effective for reducing the effect of anxiety on time perception.

## Introduction

Anxiety often seems to lengthen the perceived passage of time relative to the amount of time that is actually passing, especially when an individual is exposed to negative stimuli. Such anxiety-related time distortion has been demonstrated by many studies through different tasks. For example, Bar-Haim et al. (2010) explored the influence of negative emotion on time perception in anxiety with a time reproduction task. They found that anxious individuals have longer reproduced time for negative stimuli relative to neutral stimuli, while non-anxious individuals do not. In another study, Yoo and Lee (2015) performed a verbal estimation task to investigate the effect of emotional valence and arousal on time perception in individuals with anxiety, and observed that compared to the low-anxiety group, the high-anxiety group reported longer perceived time for high-arousal negative stimuli than for high-arousal positive, low-arousal negative, and low-arousal positive stimuli. The consistent results were also found in temporal bisection tasks (TBTs), in which participants were asked to judge if the presented time of a stimulus closed to a short standard or a long standard; the more “long” judgments a participant has, the longer the perception time (Droit-Volet et al., 2004). Jusyte et al. (2015) observed that a group of individuals with anxiety showed more “long” judgments of angry vs. neutral stimuli than did a healthy group; similarly, Ishikawa and Okubo (2016) found that participants with high anxiety showed more “long” judgments regarding negative stimuli than did those with low anxiety. This empirical evidence suggests that anxiety greatly lengthens the time perception of negative stimuli.

Although such anxiety-related time distortion has been thought to be an evolutionary basis that facilitates adaptive responses to environmental stimuli (Matthews and Meck, 2014; Lake et al., 2016), in some situations, an anxious individual might not desire such time distortion. For example, previous studies have found that prolonged waiting of time can lead to anxiety (Dasu and Rao, 2009; Van Riel et al., 2012), which in turn leads to irrational decision-making behaviors (Rajamma et al., 2009; Aniæ et al., 2011). Therefore, methods of reducing anxiety-related time overestimation are required.

The mechanism of anxiety-related time overestimation can be explained in terms of the pacemaker-accumulator (PA) model (Gibbon et al., 1984). The PA model is composed of an arousal-related pacemaker, an attention-controlled switch, and an accumulator. During the timing, the pacemaker emits pulses through the switch, and these pulses are then collected by the accumulator. The number of collected pulses represents time perception. Increasing arousal is associated with an acceleration of the pacemaker; the switch closes when attention is oriented toward timing, while the pulses are blocked when attention is oriented away from timing (Zakay and Block, 1996; Tipples, 2008; Droit-Volet, 2013; Tamm et al., 2014; Van Volkinburg and Balsam, 2014; Yoo and Lee, 2015; Lake et al., 2016; Tian et al., 2018, 2019). Theoretically, both arousal and attention can result in time distortion. Previous studies have found that anxiety would massively increase arousal when negative stimuli appear, such as increased heart rate (Shalom et al., 2015; Peng et al., 2018), increased systolic blood pressure (James et al., 1986; Matthews et al., 1986), reduced heart rate variability (Chalmers et al., 2014), excessive blushing (Gerlach et al., 2001), and increased self-report arousal (Martin, 1961; McLeod et al., 1986). Anxiety has also been found to show an abnormally large attentional bias toward negative stimuli (Bar-Haim et al., 2007), makes individual attracted by negative stimuli and difficult to distract attention from them (Yiend and Mathews, 2001; Koster et al., 2004; Pergamin-Hight et al., 2015). According to PA model, increased arousal is associated with time overestimation, while the deficit of attentional allocation toward timing is related to time underestimation, and, importantly, several studies have observed the covariant relationship between time overestimation and increased arousal that exists in anxious individuals (Jusyte et al., 2015; Yoo and Lee, 2015; Ishikawa and Okubo, 2016). Thus, the increased arousal was thought to play a dominating role in anxiety-related time overestimation (Bar-Haim et al., 2010; Jusyte et al., 2015; Yoo and Lee, 2015; Ishikawa and Okubo, 2016), and arousal-regulation methods would be most appropriate to reduce such time distortion.

In the domain of emotion regulation, reappraisal and suppression are the two most common strategies (Gross, 1998, 2007, 2014), both have been proven to be effective for regulating arousal (e.g., Eippert et al., 2007; Goldin et al., 2008; Flynn et al., 2010; Yuan et al., 2015a; Cai et al., 2016a,b), and both can be used for time perception (e.g., Effron et al., 2006; Tian et al., 2018; Uusberg et al., 2018). The reappraisal is an antecedent-focused strategy that regulates emotion by changing one’s understanding of the emotional events, while the suppression is a response-focused strategy that involves the regulation through suppressing one’s emotional expression. However, some evidence shows that suppression has a greater arousal-reducing effect than does reappraisal. Specifically, Gross (1998) compared the difference in emotion reduction between suppression and reappraisal in terms of subjective experience, expressive behavior, and physiology. The former one has been considered to be valence-related, the latter two are often used as indicators of arousal (Gross, 2007, 2014). Gross found that reappraisal would lessen the subjective experience but that suppression would not, while the reduction of expressive behavior was somewhat more pronounced for suppression than for reappraisal, and suppression showed significant reductions in physiology (i.e., finger pulse amplitude and finger temperature) but reappraisal did not. Consistent with this evidence, Gallo et al. (2012) used an implementation intention paradigm (i.e., using if-then plans to spell out when, where, and how a set goal is to be put into action “If situation x is encountered, then I will perform response y!”) to manipulate emotion regulation via reappraisal and suppression, respectively. The results showed that applying reappraisal allowed participants to consider stimuli as being less unpleasant or disgusting, but their arousal ratings were unaffected; meanwhile, participants who applied suppression reported lower evoked arousal after viewing perceptually disgusting stimuli, but their valence ratings remained unchanged. This indicates that suppression is a more effective strategy than reappraisal for reducing arousal. Thus, suppression may be an appropriate method of reducing anxiety-related time overestimation.

Suppression can be conducted either deliberately, that is, consciously based on instructions, or automatically via a priming task (e.g., a sentence-unscrambling task). The priming task is thought to passively activate the goal of emotion suppression such that it then realizes the goal without the participant’s awareness (Bargh et al., 2001). However, it has been shown that deliberate suppression of emotion, such as asking a participant “please try to avoid letting your feelings show,” often has no effect on decreasing arousal, and may even produce counterproductive results (Gross, 1998; Hofmann et al., 2009), especially for anxious individuals. For example, Campbell-Sills et al. (2006b) investigated the suppression of negative emotions in anxious participants who were instructed to perform suppression and found that the participants evidenced increased cardiac arousal. Hofmann et al. (2009) compared reappraisal, acceptance, and suppression strategies for regulating anxiety arousal, using instructions to manipulate the strategies, and found that suppressing anxiety was the least effective of the three strategies and that the suppression group’s subjective anxiety did not differ between the pre- and post-instruction stages. Similarly, Wilson et al. (2014) employed the “Swamp Metaphor,” created by Hayes et al. (1999), presenting this to participants as a strategy for addressing emotion; the researchers hoped to encourage anxious participants to suppress their emotions, but this did not produce any significant pretest–posttest change in arousal (measured via skin conductance level). Recently, Yuan et al. (2015b) found that such suppression decreases LPP amplitudes (which can be interpreted as reflecting the level of emotional arousal) during suppression vs. control conditions, but elicits larger amplitudes in central-frontal P3 (which can be interpreted as reflecting the level of attentional resource cost), suggesting that the attentional resource cost of deliberate suppression may explain why previous studies have found it a maladaptive strategy. In contrast, automatic suppression has been shown to consume few attentional resource (Mauss et al., 2007a,b; Gallo et al., 2009; Williams et al., 2009; Koole and Rothermund, 2011), and effectively reduces arousal (Mauss et al., 2007b; Chen et al., 2017b). For example, Mauss et al. (2007b) used a sentence-unscrambling task to unconsciously prime subjects of suppression or expression goals, respectively. Decreased levels of arousal were observed in emotion suppression compared to emotion expression subjects. Yang et al. (2015) found that individuals who underwent non-conscious suppression primed through a word matching task returned lower arousal levels than those in a control condition. In the physiological dimension, using a synonym-matching task to activate automatic suppression, Chen et al. (2017b) observed that the participants with greater habitual use of suppression are associated with less amygdala activity to emotional signals, which denotes the reduced intensity of responses to emotional stimuli (Chen et al., 2017a). As anxious individuals generally exist attentional resource deficits (Krug and Carter, 2010), deliberate suppression is unsuitable for regulating negative effect in participants with anxiety (Campbell-Sills et al., 2006a,b; Hofmann et al., 2009; Yin et al., 2017), whereas automatic suppression is effective in this regard (Hofmann et al., 2010). This suggests that automatic suppression, but not deliberate suppression, may be effective appropriate for reducing arousal in anxiety. Considering this, as well as the attentional resource costs and counterproductive effect of deliberate suppression, automatic suppression is likely to be effective in reducing anxiety-related time overestimation.

Consequently, the present study aimed to test the effectiveness of both deliberate suppression (Experiment 1) and automatic suppression (Experiment 2) in reducing the anxiety-related time overestimation. This study may have two important meanings: first, most previous studies have focused on using emotion regulation strategies to modify the emotional respondings (e.g., emotional valence/arousal rating, heart rate, and amygdala activity), while the present study may expand these studies to the influence of emotion regulation on time perception. Second, the present study may initiate a non-conscious emotion regulation method to modify time perception, since previous studies have mostly used conscious methods. (e.g., Mella et al., 2011; Tian et al., 2018; Uusberg et al., 2018). To select anxious participants, the trait scale of Spielberger’s State-Trait Anxiety Inventory (STAI-T, Spielberger et al., 2017) was adopted to identify individuals with anxiety. A TBT, featuring negative and neutral pictures, was used to measure time perception. Meanwhile, a self-reported nine-point scale was adopted to assess the arousal induced by each picture. Experiment 1 targeted deliberate suppression, elicited through explicit training instructions. As applying deliberate suppression to anxious individuals often results in the inability to regulate arousal (Roemer and Borkovec, 1994; Koster et al., 2003; Hofmann et al., 2009), and increased arousal is associated with time overestimation (Gibbon et al., 1984), we hypothesized that using deliberate suppression would not reduce the anxiety-related time overestimation. In Experiment 2, we activated automatic suppression by priming non-conscious suppression with an adaptation of the sentence-unscrambling task (Mauss et al., 2007b). As mentioned above, automatic suppression is effective for reducing arousal (Mauss et al., 2007b; Yang et al., 2015; Chen et al., 2017b); thus, we hypothesized that automatic suppression would effectively reduce the anxiety-related time overestimation.

## Experiment 1

### Methods

A mixed 2 × 2 design was adopted, featuring a between-subject factor of group (control, experimental) and a within-subject factor of picture type (neutral, negative).

#### Participants

We determined the sample size using an *a priori* power analysis. To detect a large effect of anxiety, using a power of 0.8 and an alpha of 0.05, the power analysis indicated a required sample size of 21 participants per group.

Fifty-two participants were recruited from a Chinese university. STAI-T showed that their anxiety severity scores range from 44 to 69. STAI-T yields a total score of between 20 and 80; there is no formal cut-off point, but scores of 40 and above are usually considered to indicate a high level of anxiety (Törnqvist et al., 2006). Participants were randomly assigned to one of two groups: a control group (18 females and eight males, mean age = 23.23 years, SD = 3.27, STAI-T score: 46–67, mean = 53.88, SD = 5.23) and an experimental group (16 females and 10 males, mean age = 22.81 years, SD = 3.44, STAI-T score: 45–65, mean = 54.12, SD = 5.62). The two groups did not differ in terms of age or STAI-T score, *p*s > 0.50; their STAI-T scores were significantly above average for Chinese college students (mean STAI-T = 45.31, SD = 11.99, *N* = 199, Li and Qian, 1995), *p*s < 0.05. All participants were right-handed and had normal or corrected-to-normal vision. All provided written informed consent before the experiment.

#### Stimuli

The stimuli used for the representation of duration were an image of a brown rectangle, 16 negative pictures, and 16 neutral pictures (size: 433 × 315 pixels). All pictures were selected from the “Chinese Affective Picture System.” The negative pictures featured frightening animals, dilapidated buildings, and disaster areas, while the neutral pictures included neutral animals, buildings, and human activities. A previous study had assessed each picture in terms of valence and arousal by asking a large sample of Chinese participants to rate them using a nine-point scale (Bai et al., 2005); an independent-samples *t*-test performed on the mean scores showed that the negative pictures differed significantly from the neutral pictures in regard to valence, *t*(30) = −21.24, *p* < 0.05 (Negative: mean = 2.74, SD = 0.32; Neutral: mean = 5.26, SD = 0.35) and arousal, *t*(30) = 20.00, *p* < 0.05 (Negative: mean = 5.78, SD = 0.48; Neutral: mean = 3.32, SD = 0.08).

#### Procedure

The experiment was conducted and recorded using E-Prime 1.1 (Psychological Software Tools, Pittsburgh, PA, United States) on a PC. Stimuli were presented on a 17” LCD monitor (1024 × 768 pixels, 60 Hz), and the participants gave their responses using the computer keyboard. The participants were seated in a quiet room approximately 60 cm from the monitor, with horizontal and vertical visual angles of less than 16°.

**FIGURE 1:**
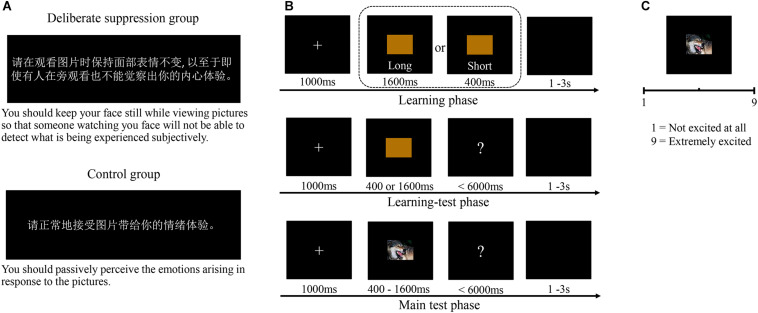
The procedure of Experiment 1. **(A)** The instructions of deliberate suppression group and control group. **(B)** Schematic illustration of the temporal bisection task. **(C)** Schematic illustration of the arousal assessment.

The experimental group and the control group were presented with different instructions before they performed the TBT. The experimental group was trained to suppress their expressions of emotion and to deliberately stay calm when a negative picture appeared. The control group, meanwhile, were trained to passively perceive the emotions they felt in response to the pictures they were shown (see Figure 1A).

Both groups were given the same TBT, consisting of a learning, a learning test, and a main test phase. In the learning phase, a brown rectangle appeared for a short (400 ms) or long (1600 ms) standard duration (five times each, random order). Participants were asked to remember these durations. In the learning test, participants were instructed to indicate whether stimuli appeared for either a short (400 ms) or a long (1600 ms) duration (five times each, random order). Participants whose accuracy was less than 100% would learn again. In the main test phase, the 16 negative and 16 neutral pictures were randomly presented with seven durations (400, 600, 800, 1000, 1200, 1400, and 1600 ms; see Figure 1B). Each picture was presented once for each duration, meaning there were 224 trials altogether. Participants were asked to indicate, by pressing one of two labeled keys (“j” and “f,” counterbalanced for long/short) on the keyboard, whether each picture appeared for a duration closer to either the “short” or the “long” standard duration.

After the TBT, participants were required to assess the arousal of each picture using a nine-point scale (from 1 = “not excited at all” to 9 = “extremely excited”; see Figure 1C). The experimental group and the control group were also required to deliberately suppress or passively perceive emotions during the arousal assessment.

Finally, participants were required to rate the extent to which they deliberately suppressed (experimental group) or passively perceived (control group) emotion using a nine-point scale (from 1 = “not at all” to 9 = “completely”). The rating scores ranged from 7 to 9 (control group: mean = 8.50, SD = 0.81; experimental group: mean = 8.42, SD = 0.81), suggesting that the manipulation of deliberate suppression in the experimental and passive perception in the control groups were both successful.

The experimental protocol was approved by the local ethics committee.

### Results

The raw data of TBT were analyzed by computing the proportion of “long” responses in the TBT. For each duration (t), the corresponding proportion of “long” responses was computed as P(“long”| t). A plot of proportions revealed a psychometric function that took the form of a sigmoid curve extending from approximately 0, representing the shortest duration, to approximately 1, representing the longest. The function shift toward the left indicates that the participants perceived longer time. The resulting curves were fitted using a cumulative Gaussian function with two parameters: (1) the standard deviation (σ), corresponding to temporal sensitivity, and (2) the mean (μ), corresponding to the “point of subject equality” (PSE), or the duration that yielded no difference between the long and short responses [i.e., P(long| t = μ) = 0.5] (Kroger-Costa et al., 2013; Liu et al., 2015; Tian et al., 2018). A lower PSE indicates that the participants perceived longer time.

**FIGURE 2:**
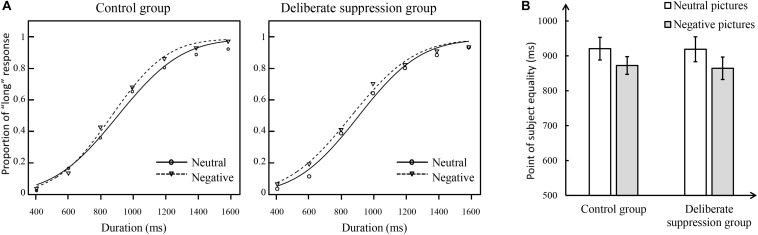
The temporal results of Experiment 1. **(A)** Proportion of “long” responses plotted against durations ranging between 400 and 1600 ms for the control group (left panel) and deliberate suppression group (right panel) by the negative and neutral pictures. **(B)** Mean point of subjective equality for neutral and negative pictures in each group. The error bar represents the standard error.

Figure 2A shows the proportion of “long” responses plots of Experiment 1. It shows that the psychophysical functions were shifted toward the left, consistent with a time overestimation, for the negative pictures compared to the neutral pictures in both control and experimental groups. To statistically analyze the effect of deliberate suppression on time perception, a repeated-measures ANOVA was run on PSE, with group (control, experimental) as a between-subject factor, and picture type (neutral, negative) as a within-subject factor; this revealed a significant main effect of picture type, *F*(1,50) = 10.14, *p* < 0.01, η*_*p*_*^2^ = 0.17. *Post hoc* analysis revealed that PSE was systematically lower for the negative pictures (mean ± SE = 868.39 ± 20.81 ms) than for the neutral pictures (919.67 ± 24.14 ms). In contrast, the main effect of group, *F*(1,50) = 0.01, *p* = 0.91, and the interaction between group and picture type, *F*(1,50) = 0.04, *p* = 0.85, was non-significant. Thus, no effect of deliberate suppression on reducing anxiety-related time distortion was observed in the ANOVA (see Figure 2B).

However, as a group design, the between-subject factors may influence results (Penney et al., 2000). To further evaluate the effect of deliberate suppression on anxiety-related time overestimation, we used PSEs of neutral pictures and negative pictures to calculate the overestimation of each participant (PSE_*overestimated*_ = PSE_*neutral*_ – PSE_*negative*_). An independent-samples *t*-test run on PSE_*overestimated*_ revealed that PSE_*overestimated*_ did not significantly differ between the experimental group and control group, *t*(50) = 0.20, *p* = 0.85. This suggests that deliberate suppression could not reduce anxiety-related time overestimation.

**FIGURE 3:**
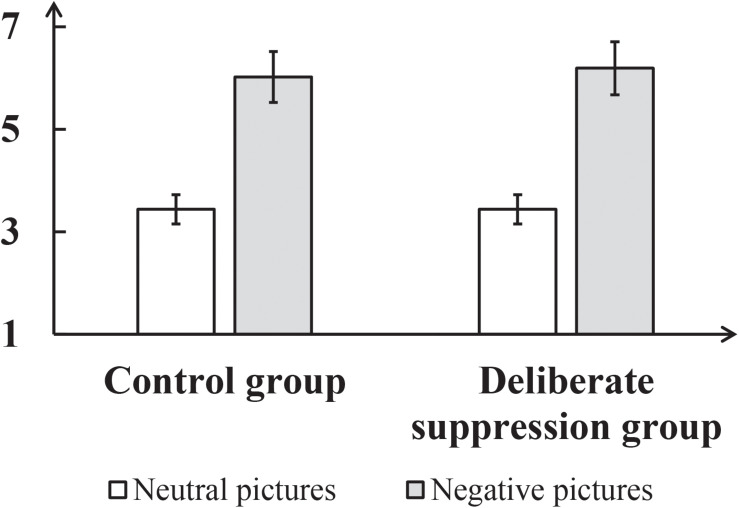
Mean self-reported arousal for neutral and negative pictures in each group in Experiment 1. The error bar represents the standard deviation.

In addition, self-reported arousal was analyzed. To test the effect of deliberate suppression on arousal, a repeated-measures ANOVA was performed on arousal rating, with group (control, experimental) as a between-subject factor and picture type (neutral, negative) as a within-subject factor (see Figure 3). This only revealed a significant main effect of picture type, *F*(1,50) = 1312.15, *p* < 0.001, η*_*p*_*^2^ = 0.96. The *post hoc* analysis revealed that arousal was systematically higher for the negative pictures (6.11 ± 0.07) than for the neutral pictures (3.44 ± 0.04).

To avoid the potential effect of individual difference, we used each participants’ arousal concerning neutral pictures and those concerning negative pictures to calculate their “arousal-increase” (Arousal_*increase*__*d*_ = Arousal_*negative*_ – Arousal_*neutral*_). An independent-samples *t*-test showed that Arousal_*increase*__*d*_ did not significantly differ between the experimental and control groups, *p* = 0.30. Taken together, this indicates that, for anxious individuals, deliberate suppression has no effect on the arousal induced by negative pictures.

### Discussion

In Experiment 1, we tested whether deliberate suppression reduces anxiety-related time overestimation. To confirm our hypothesis regarding Experiment 1, we assessed the effect of deliberate suppression using both PSE and PSE_*overestimated*_. In accordance with our hypothesis, the results of time perception showed that giving instructions regarding deliberate suppression did not reduce the experimental participants’ overestimation regarding the PSE of the negative pictures to be any lower than that of the control group. This indicates that deliberate suppression is not an appropriate way to reduce the tendency of individuals with anxiety to engage in massive time overestimation of negative stimuli.

Additional analyses of self-reported arousal found that deliberate suppression did not reduce arousal in response to negative pictures compared with the control group. Since increased arousal plays a dominating role in the anxiety-related time overestimation (Jusyte et al., 2015; Yoo and Lee, 2015; Ishikawa and Okubo, 2016), it is, therefore, inferred that the failure of deliberate suppression to reduce arousal in the anxious group may account for the lack of effect on anxiety-related time overestimation.

It should be noted that these results are not completely consistent with previous studies, which have found deliberate suppression results in counterproductive increasing arousal (e.g., Gross, 1998; Campbell-Sills et al., 2006b; Hofmann et al., 2009). A reasonable explanation is as follows: deliberate suppression consumes attentional resource (Yuan et al., 2015b), but anxious individuals generally exhibit attentional resource deficits (Krug and Carter, 2010). Since the present main task is timing, the anxious individuals did not have enough resources to realize the goal of emotional suppression, which resulted in a null effect. Another explanation lies in that all our participants are East Asians. Previous studies have shown that East Asians perform better than Westerners in suppression (Butler et al., 2007; Murata et al., 2013), and the suppression produces beneficial emotion-regulation effects at both behavioral and physiological levels for East Asians (Yuan et al., 2015b), so it is likely that anxious East Asians could exhibit lesser negative effects of suppression (i.e., counterproductive increasing arousal) than Westerners. Nonetheless, the results of the present study and previous studies have consistently shown that deliberate suppression is not an effective strategy for reducing arousal in anxiety.

Therefore, an attentional resource-costly regulation method such as deliberate suppression may not apply to anxious individuals, who are characterized by deficits in cognitive control mechanisms (Krug and Carter, 2010). This implies that non-conscious emotion regulation (e.g., automatic suppression), which requires no deliberate cognitive effort during negative stimulation, may better reduce the anxiety-related time distortion.

## Experiment 2

### Methods

A mixed 2 × 2 design was adopted, featuring a between-subject factor of group (control, experimental) and a within-subject factor of picture type (neutral, negative).

#### Participants

A new cohort of 52 participants from the same Chinese university was recruited for Experiment 2. They were randomly assigned to one of two groups: a control group (17 females and nine males, mean age = 23.88 years, SD = 3.31, STAI-T score = 44–68, mean = 54.92, SD = 5.28) and an experimental group (15 females and 11 males, mean age = 24.31 years, SD = 3.33, STAI-T score = 45–66, mean = 55.27, SD = 5.45). The two groups did not differ in regard to age or STAI-T (*p*s > 0.05), and their STAI-T scores were significantly above average for Chinese college students, *p*s < 0.05. All participants were right-handed and had normal or corrected-to-normal vision. They gave written informed consent before the experiment.

#### Stimuli

The stimuli were the same as those used in Experiment 1.

#### Procedure

**FIGURE 4:**
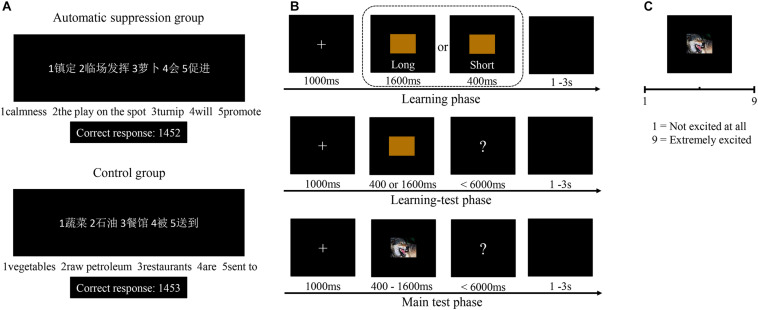
The procedure of Experiment 2. **(A)** Schematic illustration of the sentence-unscrambling task for the automatic suppression group and control group. Each sentence unscrambling task contains five Chinese words. **(B)** Schematic illustration of the temporal bisection task. **(C)** Schematic illustration of the arousal assessment.

The experimental devices and the procedure for Experiment 2 were similar to that used in Experiment 1, except we administered sentence-unscrambling tasks instead of providing instructions before the TBT (see Figure 4). The sentence-unscrambling task was used to prime participants of suppression automatically. It was first adapted to manipulate automatic emotion regulation by Mauss et al. (2007b). In this task, participants are asked to construct grammatically correct, four-word sentences from five-word jumbles; 40 sentences including either passive perception or emotion-suppression terms were used to prime the control group and experimental group, respectively (see Figure 4A). These terms were chosen by asking 15 postgraduate students (seven males) to “list the 10 words that come to your mind when you think of the concepts ‘emotion suppression’ or ‘passively perceiving’.” Then, five new postgraduate students majoring in emotion regulation evaluated these sentences and selected 20 sentences each that served to prime suppression or passive perception. To avoid potential demand effects, we informed participants that the sentence-unscrambling task was designed to test their grammatical abilities.

At the end of Experiment 2, participants were required to complete a funneled debriefing procedure, which is similar to previous studies (e.g., Chartrand and Bargh, 1996; Bargh and Chartrand, 2000; Williams et al., 2009). They were asked (1) whether they had ever seen or completed a sentence-unscrambling task for another experiment, (2) what they thought the purpose of the sentence-unscrambling task, (3) whether and what they thought the sentence-unscrambling task and TBT had been related. This funneled debriefing procedure was used to check the validity of the automatic suppression manipulation. For the first question, nobody reported they had seen or completed a sentence-unscrambling task before. For the second question, most participants(*n* = 47) believed that the purpose of the sentence-unscrambling task was to test their grammatical abilities, and a few participants (*n* = 5) reported that they did not know. For the third question, nearly half of the participants (*n* = 24) thought that the sentence-unscrambling task had nothing to do with TBT, and the remaining participants thought that the sentence-unscrambling task may have some relationship with TBT (*n* = 28), but nobody mentioned emotion or emotion regulation as the connection between the two tasks (*n* = 0). Thus, no participants indicated suspicion of the prime or guessed the hypothesis of the experiment.

The experimental protocol was approved by the local ethics committee.

### Results

**FIGURE 5:**
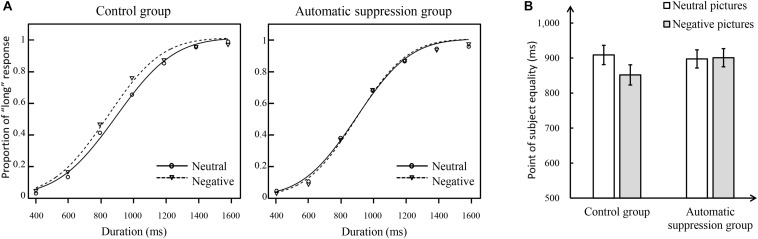
The temporal results of Experiment 2. **(A)** Proportion of “long” responses plotted against durations ranging between 400 and 1600 ms for the control group (left panel) and automatic suppression group (right panel) by the negative and neutral pictures. **(B)** Means point of subjective equality for neutral and negative pictures in each group. The error bar represents the standard error.

Figure 5A shows the proportion of “long” responses plots of Experiment 2. It shows that the psychophysical functions were shifted toward the left, consistent with a time overestimation, for the negative pictures compared to the neutral pictures only in the control group. To statistically analyze the effect of automatic suppression on time perception, a repeated-measures ANOVA was performed on PSE, with group (control, experimental) as a between-subject factor and picture type (neutral, negative) as a within-subject factor; this revealed a significant main effect of picture type, *F*(1,50) = 4.47, *p* < 0.05, η*_*p*_*^2^ = 0.08. *Post hoc* analysis revealed that PSE was lower for negative pictures (876.27 ± 19.42 ms) than for neutral pictures (903.11 ± 18.92 ms); in contrast, the main effect of group did not reach significance, *F*(1,50) = 0.27, *p* > 0.05. Importantly, the interaction between group and picture type was significant, *F*(1,50) = 5.66, *p* < 0.05, η*_*p*_*^2^ = 0.10. A simple effects analysis showed that in the control group, the PSE of negative pictures was lower than that of neutral pictures, *p* < 0.01, whereas in the experimental group, there was no difference, *p* = 0.85, indicating that the anxiety-related time overestimation in the control group did not emerge in the experimental group (see Figure 5B).

To further evaluate the effect of automatic suppression on reducing anxiety-related time overestimation, we also used the PSE of neutral pictures and of negative pictures to calculate the overestimation of each participant (PSE_*overestimated*_ = PSE_*neutral*_ – PSE_*negative*_). An independent-samples *t*-test showed that the PSE_*overestimated*_ of the experimental group was significantly lower than that of the control group, *t*(50) = 2.38, *p* < 0.05, Cohen’s *d* = 0.66. This result indicates that the automatic suppression could reduce the anxiety-related time overestimation.

**FIGURE 6:**
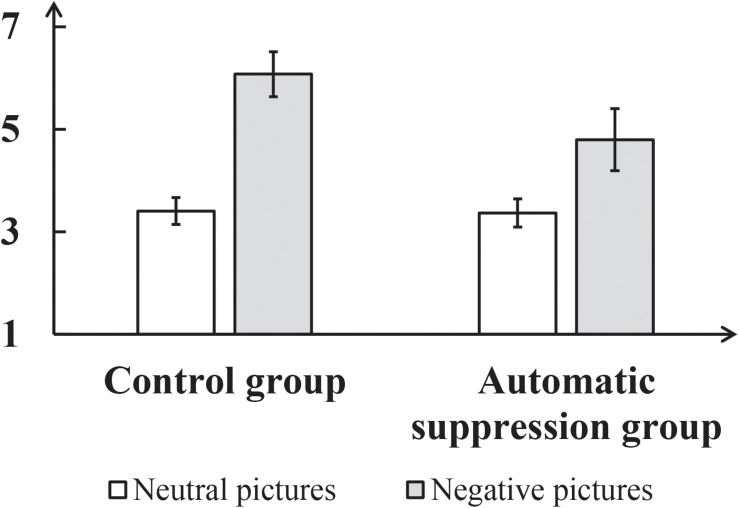
Mean self-reported arousal for neutral and negative pictures in each group in Experiment 2. The error bar represents the standard deviation.

In addition, self-reported arousal was analyzed. To test the effect of automatic suppression on arousal, a repeated-measures ANOVA was conducted on arousal, with group (control, experimental) as a between-subject factor and picture type (neutral, negative) as a within-subject factor (see Figure 6). This revealed a significant interaction between group and picture type, *F*(1,50) = 9.96, *p* < 0.001, η*_*p*_*^2^ = 0.57. A simple effect analysis showed that in the experimental group the level of arousal induced by the negative pictures (4.80 ± 0.10) was lower than that in the control group (6.07 ± 0.10), *p* < 0.001. Meanwhile, for the neutral pictures, the level of arousal shown by the experimental group (3.37 ± 0.05) did not significantly differ from that shown by the control group (3.41 ± 0.05), *p* = 0.59.

To avoid the potential effect of individual difference, we also used the arousal levels of the neutral and negative pictures to calculate each participant’s “arousal-increase” (Arousal_*increase*__*d*_ = Arousal_*negative*_ − Arousal_*neutral*_). An independent-samples *t*-test showed that the arousal-difference of the experimental group (1.43 ± 0.11) was lower than that of the control group (2.67 ± 0.10), *t*(50) = 8.14, *p* < 0.001, Cohen’s *d* = 2.27. Taken together, this indicates that, for anxious individuals, automatic suppression could reduce the arousal induced by negative stimuli.

Furthermore, we tested the relationship between time overestimation and arousal reduction. A Pearson’s correlation coefficient was conducted, in which PSE_*overestimated*_ was used as time overestimation data and Arousal_*increase*__*d*_ was used as arousal-reduction data. The results showed that Arousal_*increase*__*d*_ positively correlated with PSE_*overestimated*_, *r* = 0.53, *p* < 0.001, indicating that the more arousal was reduced, the less time overestimation occurred.

Moreover, we performed a mediation analysis with the PROCESS macro developed by Hayes (2013) to examine whether the relationship between automatic suppression (X) and PSE_*overestimated*_ (Y) was mediated by Arousal_*increase*__*d*_ (M). Y and M were transformed into z scores. The results showed that the total effect of X on Y was significant, *B* = −0.63, SE = 0.27, 95% CI, [−1.16, −0.10], *t* = −2.38, *p* < 0.05, the effect of X on M was significant, *B* = −1.50, SE = 0.18, 95% CI, [−1.86, −1.13], *t* = −8.14, *p* < 0.001, the effect of M on Y was significant, *B* = 0.66, SE = 0.18, 95% CI, [0.30, 1.03], *t* = 3.63, *p* < 0.001, the indirect effect through Arousal_*increase*__*d*_ was significant, β = −0.99, SE = 0.21, 95% CI, [−1.38, −0.54], but the direct effect of X on Y was not significant, β = 0.36, SE = 0.36, 95% CI, [−0.37, 1.09], *t* = 1.00, *p* = 0.32, thus indicating that in Arousal_*increase*__*d*_ acts as mediator between automatic suppression and PSE_*overestimated*_.

### Discussion

In Experiment 2, we tested whether automatic suppression can reduce time overestimation in anxious individuals using both PSE and PSE_*overestimated*_. In accordance with our hypothesis, the results showed that using a sentence-unscrambling task with automatic suppression caused the participants’ overestimation of the perceived duration of the negative pictures to be less than that of the control group.

An additional analysis of self-reported arousal found that automatic suppression could reduce the arousal level induced by negative pictures, and that a reduction in time overestimation positively correlated with a reduction in arousal. Importantly, a mediation analysis revealed that the effect of automatic suppression on anxiety-related time overestimation was moderated by the arousal reduction. These results imply that automatic suppression, which effectively reduces arousal (Gross, 1998; Gallo et al., 2012), is an appropriate method for reducing anxiety-related time overestimation.

## General Discussion

The present study focused on finding an effective way of reducing anxiety-related time overestimation. As increased arousal has been thought to be the main mechanism of the anxiety-related time overestimation (Jusyte et al., 2015; Yoo and Lee, 2015; Ishikawa and Okubo, 2016), we adopted suppression, which is effective for reducing arousal, as a possible method. Because suppression can be achieved both deliberately and automatically, Experiments 1 and 2 were tested the effectiveness of deliberate suppression and automatic suppression in reducing anxiety-related time overestimation, respectively.

The results for the control groups in both Experiments 1 and 2 replicated those of previous studies, with anxious individuals showing longer perceived durations of negative pictures than of neutral pictures (Bar-Haim et al., 2010; Jusyte et al., 2015; Yoo and Lee, 2015; Ishikawa and Okubo, 2016). This means that the time overestimation of negative pictures was successfully replicated, providing a baseline for investigating the effects of deliberate and automatic suppression on this time distortion.

As hypothesized, in Experiment 1 deliberate suppression failed to reduce the time overestimation in anxious individuals, with non-significant arousal reduction being observed. This result is inconsistent with the findings of a previous study based on healthy individuals (Tian et al., 2018). In contrast, Experiment 2 showed that automatic suppression successfully reduced anxiety-related time overestimation with significant arousal reduction. The inconsistent results between Experiments 1 and 2 suggest that the manner in which suppression is performed (deliberate/automatic) influences the effect of suppression. Previous studies have suggested that deliberate suppression consumes considerable attentional resource (Yuan et al., 2015b; Cai et al., 2016a,b). Individuals with psychological disorders (e.g., anxiety) usually experience a shortage of attentional resource as a result of regularly engaging in effortful and controlled processes based on deliberate suppression (Gao et al., 2018). This deficiency presumably explains the failure of deliberate suppression in Experiment 1. In contrast, as automatic suppression consumes little or no attentional resource, Experiment 2 showed that automatic suppression causes a successful modulation effect of anxiety-related time overestimation. Thus, we can surmise that unawareness of suppression may be the key to its effectiveness.

Since increased arousal plays a dominating role in anxiety-related time overestimation (Jusyte et al., 2015; Yoo and Lee, 2015; Ishikawa and Okubo, 2016), it is likely to infer that the effectiveness of suppression to reduce the time overestimation is related with the effectiveness of arousal reduction. Several studies have shown that awareness of suppression usually does not reduce arousal, and may even lead to counterproductive increases in arousal, especially for anxious individuals (e.g., Campbell-Sills et al., 2006b; Hofmann et al., 2009; Wilson et al., 2014). Meanwhile, other studies have indicated that unawareness of suppression leads to decreased arousal (e.g., Mauss et al., 2007b; Williams et al., 2009; Chen et al., 2017b). These support the current finding that automatic suppression, which is less cognitively taxing, is more effective than deliberate suppression in terms of reducing anxiety-related time overestimation.

The present study extends the non-conscious emotion regulation research. In previous studies, researchers focused on the effect of non-conscious emotion regulation on the emotional responding to emotion events. For example, Mauss et al. (2007b) primed “emotion control” goals to investigate the effect of automatic suppression on participants’ self-reported anger experience and corresponding cardiovascular responding; Williams et al. (2009) primed reappraisal goals to assess the effect of automatic reappraisal on heart rate during stress. By contrast, the present study aimed at the effect of non-conscious emotion regulation on time perception, which is a cognitive function that could be, however, affected by emotion. The present study also extends the research of time perception modifications. In previous studies, researchers have used the conscious methods to modify time perception, such as deliberate suppression, reappraisal (Tian et al., 2018), appraisal (Uusberg et al., 2018), or attention allocation (Mella et al., 2011); instead, the present study initiated a non-conscious method to modify time perception. The present findings revealed that automatic suppression is an effective way to reduce anxiety-related time overestimation.

Several limitations to the present study should be addressed in future work. First, we only employed Chinese participants. The efficacy of suppression is culture-specific: East Asians show better performance than Westerners (Butler et al., 2007; Murata et al., 2013). In addition, in Chinese people suppression has been determined to produce beneficial emotion-regulation effects at both behavioral and physiological levels (Yuan et al., 2015b). Therefore, for Westerners, suppression might be less effective for reducing anxiety-related time overestimation; further studies should aim to verify this. Second, we adopted a self-reported nine-point scale to assess participants’ arousal. Some studies pointed out that self-reported assessment is not as precise as the physiological measurement (Wilhelm et al., 2001; Edelmann and Baker, 2002; Mauss et al., 2004), although self-reporting and physiological results are somewhat consistent (McLeod et al., 1986; Gross, 1998; Kuo and Linehan, 2009). Thus, if future studies need to accurately explore the relationship between arousal and time perception, physiological measurements should be used. Third, we did not directly compare the effect between deliberate and automatic suppression as their manipulations didn’t match in the time design level. Future studies could design an experiment that includes both conditions, so that their effects could be compared. Fourth, we only tested the effect of suppression and ignored other strategies. Previous studies have found that not only suppression (Effron et al., 2006; Tian et al., 2018), but also attention allocation (Mella et al., 2011), reappraisal (Tian et al., 2018), and conscious awareness of time distortion (Droit-Volet et al., 2015) are effective modifications of time distortion—especially attention allocation, which regulates negative emotions more rapidly than does suppression (Paul et al., 2013), protects individuals from mood disorders during frustrating situations (Li and Yuan, 2018), and maintains positive affect and well-being during negative stimulation (Yuan et al., 2012; Meng et al., 2015). Future studies might explore these and other strategies for modifying anxiety-related time overestimation.

## Conclusion

Increasingly, researchers are searching for methods of modifying time distortion (Effron et al., 2006; Mella et al., 2011; Droit-Volet et al., 2015; Tian et al., 2018; Uusberg et al., 2018). Previous works have mostly focused on healthy individuals. However, anxious individuals are associated with more time overestimation than do healthy individuals (Bar-Haim et al., 2010; Jusyte et al., 2015; Yoo and Lee, 2015; Ishikawa and Okubo, 2016). Thus, reducing anxiety-related time overestimation is an emerging challenge.

The anxiety-related time overestimation is mainly associated with increased arousal (Jusyte et al., 2015; Yoo and Lee, 2015; Ishikawa and Okubo, 2016). The present study employed deliberate suppression and automatic suppression, which both have been proven to be an effective strategy for reducing arousal, as methods of modifying anxiety-related time overestimation. The experiments performed in this work initiated empirical evidence that anxiety-related time overestimation can be effectively reduced by automatic suppression, but not deliberate suppression.

## Data Availability Statement

All datasets generated for this study are included in the article/Supplementary Material.

## Ethics Statement

The studies involving human participants were reviewed and approved by the ethics committee of Southwest University. The patients/participants provided their written informed consent to participate in this study.

## Author Contributions

JY and YT designed the experiments. YT and LL acquired and analyzed the data. All authors contributed to the interpretation of the data and approved the final version of the manuscript.

## Conflict of Interest

The authors declare that the research was conducted in the absence of any commercial or financial relationships that could be construed as a potential conflict of interest.

## References

[B1] Aniæ I. D. Radas S. Miller J. C. (2011). Antecedents of consumers’ time perceptions in a hypermarket retailer. *Serv. Ind. J.* 31 809–828. 10.1080/02642060903067530

[B2] Bai L. Ma H. Huang Y. Luo Y. (2005). The development of native chinese affective picture system—a pretest in 46 college students. *Chinese Ment. Heal. J.* 19 719–722. 10.3321/j.issn:1000-6729.2005.11.001 30704229

[B3] Bargh J. A. Chartrand T. L. (2000). “The mind in the middle: a guide to priming and automaticity resaerch,” in *Handbook of Research Methods in Social and Personality Psychology*, eds Reis H. T. Judd C. M. (Cambridge: Cambridge University Press), 253–285.

[B4] Bargh J. A. Gollwitzer P. M. Lee-Chai A. Barndollar K. Trötschel R. (2001). The automated will: nonconscious activation and pursuit of behavioral goals. *J. Pers. Soc. Psychol.* 81 1014–1027. 10.1037/0022-3514.81.6.1014 11761304 PMC3005626

[B5] Bar-Haim Y. Kerem A. Lamy D. Zakay D. (2010). When time slows down: the influence of threat on time perception in anxiety. *Cogn. Emot.* 24 255–263. 10.1080/02699930903387603

[B6] Bar-Haim Y. Lamy D. Pergamin L. Bakermans-Kranenburg M. J. Van Ijzendoorn M. H. (2007). Threat-related attentional bias in anxious and nonanxious individuals: a meta-analytic study. *Psychol. Bull.* 133 1–24. 10.1037/0033-2909.133.1.1 17201568

[B7] Butler E. A. Lee T. L. Gross J. J. (2007). Emotion regulation and culture?: are the social consequences of emotion suppression emotion regulation and culture?: are the social consequences of emotion suppression culture-specific? *Emotion* 7:30. 10.1037/1528-3542.7.1.30 17352561

[B8] Cai A. Lou Y. Long Q. Yuan J. (2016a). The sex differences in regulating unpleasant emotion by expressive suppression: extraversion matters. *Front. Psychol.* 7:1011. 10.3389/fpsyg.2016.01011 27458408 PMC4935688

[B9] Cai A. Yang J. Xu S. Yuan J. (2016b). The male advantage in regulating negative emotion by expressive suppression: an event-related potential study. *Acta Psychol. Sin.* 48:482 10.3724/SP.J.1041.2016.00482

[B10] Campbell-Sills L. Barlow D. H. Brown T. A. Hofmann S. G. (2006a). Acceptability and suppression of negative emotion in anxiety and mood disorders. *Emotion* 6 587–595. 10.1037/1528-3542.6.4.587 17144750

[B11] Campbell-Sills L. Barlow D. H. Brown T. A. Hofmann S. G. (2006b). Effects of suppression and acceptance on emotional responses of individuals with anxiety and mood disorders. *Behav. Res. Ther.* 44 1251–1263. 10.1016/j.brat.2005.10.001 16300723

[B12] Chalmers J. A. Quintana D. S. Abbott M. J. A. Kemp A. H. (2014). Anxiety disorders are associated with reduced heart rate variability: a meta-analysis. *Front. Psychiatry* 5:80. 10.3389/fpsyt.2014.00080 25071612 PMC4092363

[B13] Chartrand T. L. Bargh J. A. (1996). Automatic activation of impression formation and memorization goals: nonconscious goal priming reproduces effects of explicit task instructions. *J. Pers. Soc. Psychol.* 71 464–478. 10.1037/0022-3514.71.3.464

[B14] Chen S. Chen C. Yang J. Yuan J. (2017a). Trait self-consciousness predicts amygdala activation and its functional brain connectivity during emotional suppression: an fMRI analysis. *Sci. Rep.* 7:117. 10.1038/s41598-017-00073-3 28273918 PMC5428331

[B15] Chen S. Deng Z. Xu Y. Long Q. Yang J. Yuan J. (2017b). Individual differences in spontaneous expressive suppression predict amygdala responses to fearful stimuli: the role of suppression priming. *Front. Psychol.* 8:1. 10.3389/fpsyg.2017.00001 28197108 PMC5281595

[B16] Dasu S. Rao J. (2009). A dynamic process model of dissatisfaction for unfavorable, non-routine service encounters. *Prod. Oper. Manag.* 8 282–300. 10.1111/j.1937-5956.1999.tb00309.x

[B17] Droit-Volet S. (2013). Time perception, emotions and mood disorders. *J. Physiol. Paris* 107 255–264. 10.1016/j.jphysparis.2013.03.005 23542546

[B18] Droit-Volet S. Brunot S. Niedenthal P. (2004). BRIEF REPORT perception of the duration of emotional events. *Cogn. Emot.* 18 849–858. 10.1080/02699930341000194

[B19] Droit-Volet S. Lamotte M. Izaute M. (2015). The conscious awareness of time distortions regulates the effect of emotion on the perception of time. *Conscious. Cogn.* 38 155–164. 10.1016/j.concog.2015.02.021 25890486

[B20] Edelmann R. J. Baker S. R. (2002). Self-reported and actual physiological responses in social phobia. *Br. J. Clin. Psychol.* 41 1–14. 10.1348/014466502163732 11931674

[B21] Effron D. A. Niedenthal P. M. Gil S. Droit-Volet S. (2006). Embodied temporal perception of emotion. *Emotion* 6 1–9. 10.1037/1528-3542.6.1.1 16637745

[B22] Eippert F. Veit R. Weiskopf N. Erb M. Birbaumer N. Anders S. (2007). Regulation of emotional responses elicited by threat−related stimuli. *Hum. Brain Mapp.* 28 409–423. 10.1002/hbm.20291 17133391 PMC6871321

[B23] Flynn J. J. Hollenstein T. Mackey A. (2010). The effect of suppressing and not accepting emotions on depressive symptoms: is suppression different for men and women? *Pers. Individ. Diff.* 49 582–586. 10.1016/j.paid.2010.05.022

[B24] Gallo I. S. Keil A. McCulloch K. C. Rockstroh B. Gollwitzer P. M. (2009). Strategic automation of emotion regulation. *J. Pers. Soc. Psychol.* 96:11. 10.1037/a0013460 19210061

[B25] Gallo I. S. McCulloch K. C. Gollwitzer P. M. (2012). Differential effects of various types of implementation intentions on the regulation of disgust. *Soc. Cogn.* 30 1–17. 10.1521/soco.2012.30.1.1

[B26] Gao W. Chen S. Long Q. Yang J. Yuan J. (2018). The progress of emotion regulation methods and paradigms: from voluntary emotion regulation to automatic emotion regulation. *Chinese Sci. Bull.* 63 415–424. 10.1360/N972017-00727

[B27] Gerlach A. L. Wilhelm F. H. Gruber K. Roth W. T. (2001). Blushing and physiological arousability in social phobia. *J. Abnorm. Psychol.* 110 247–258. 10.1037/0021-843X.110.2.247 11358019

[B28] Gibbon J. Church R. M. Meck W. H. (1984). Scalar timing in memory. *Ann. N. Y. Acad. Sci.* 423 52–77. 10.1111/j.1749-6632.1984.tb23417.x 6588812

[B29] Goldin P. R. McRae K. Ramel W. Gross J. J. (2008). The neural bases of emotion regulation: reappraisal and suppression of negative emotion. *Biol. Psychiatry* 63 577–586. 10.1016/j.biopsych.2007.05.031 17888411 PMC2483789

[B30] Gross J. J. (1998). Antecedent-and response-focused emotion regulation: divergent consequences for experience, expression, and physiology. *J. Pers. Soc. Psychol.* 74:224. 10.1037/0022-3514.74.1.224 9457784

[B31] Gross J. J. (2007). *Handbook of Emotion Regulation.* New York, NY: Guilford Press.

[B32] Gross J. J. (2014). *Handbook of Emotion Regulation*, 2nd Edn New York, NY: Guilford Press.

[B33] Hayes S. C. Strosahl K. D. Wilson K. G. (1999). *Acceptance and Commitment Therapy.* New York, NY: Guilford Press.

[B34] Hayes A. F. (2013). *Introduction to Mediation, Moderation, and Conditional Process Analysis: A Regression-based Approach.* New York, NY: Guilford Press.

[B35] Hofmann S. G. Heering S. Sawyer A. T. Asnaani A. (2009). How to handle anxiety: the effects of reappraisal, acceptance, and suppression strategies on anxious arousal. *Behav. Res. Ther.* 47 389–394. 10.1016/j.brat.2009.02.010 19281966 PMC2674518

[B36] Hofmann S. G. Sawyer A. T. Witt A. A. Oh D. (2010). The effect of mindfulness-based therapy on anxiety and depression: a meta-analytic review. *J. Consult. Clin. Psychol.* 78:169. 10.1037/a0018555 20350028 PMC2848393

[B37] Ishikawa K. Okubo M. (2016). Overestimation of the subjective experience of time in social anxiety: effects of facial expression, gaze direction, and time course. *Front. Psychol.* 7:611. 10.3389/fpsyg.2016.00611 27199844 PMC4850902

[B38] James G. D. Yee L. S. Harshfield G. A. Blank S. G. Pickering T. G. (1986). The influence of happiness, anger, and anxiety on the blood pressure of borderline hypertensives. *Psychosom. Med*. 48 502–508. 10.1097/00006842-198609000-00005 3763789

[B39] Jusyte A. Schneidt A. Schönenberg M. (2015). Temporal estimation of threatening stimuli in social anxiety disorder: investigation of the effects of state anxiety and fearfulness. *J. Behav. Ther. Exp. Psychiatry* 47 25–33. 10.1016/j.jbtep.2014.11.006 25462982

[B40] Koole S. L. Rothermund K. (2011). “I feel better but I don’t know why”: the psychology of implicit emotion regulation. *Cogn. Emot.* 25 389–399. 10.1080/02699931.2010.550505 21432681

[B41] Koster E. H. W. Crombez G. Verschuere B. De Houwer J. (2004). Selective attention to threat in the dot probe paradigm: differentiating vigilance and difficulty to disengage. *Behav. Res. Ther.* 42 1183–1192. 10.1016/j.brat.2003.08.001 15350857

[B42] Koster E. H. W. Rassin E. Crombez G. Näring G. W. B. (2003). The paradoxical effects of suppressing anxious thoughts during imminent threat. *Behav. Res. Ther.* 41 1113–1120. 10.1016/S0005-7967(03)00144-X 12914812

[B43] Kroger-Costa A. Machado A. Santos J. A. (2013). Effects of motion on time perception. *Behav. Processes* 95 50–59. 10.1016/j.beproc.2013.02.002 23454436

[B44] Krug M. K. Carter C. S. (2010). Adding fear to conflict: a general purpose cognitive control network is modulated by trait anxiety. *Cogn. Affect. Behav. Neurosci.* 10 357–371. 10.3758/CABN.10.3.357 20805537

[B45] Kuo J. R. Linehan M. M. (2009). Disentangling emotion processes in borderline personality disorder: physiological and self-reported assessment of biological vulnerability, baseline intensity, and reactivity to emotionally evocative stimuli. *J. Abnorm. Psychol.* 118 531–544. 10.1037/a0016392 19685950 PMC4277483

[B46] Lake J. I. LaBar K. S. Meck W. H. (2016). Emotional modulation of interval timing and time perception. *Neurosci. Biobehav. Rev.* 64 403–420. 10.1016/j.neubiorev.2016.03.003 26972824 PMC5380120

[B47] Li H. Yuan J. (2018). The emotion regulation effect of unconscious distraction on the subclinical depression. *Chinese Sci. Bull.* 63 1–14. 10.1360/N972017-01253

[B48] Li W. Qian M. (1995). The Chinese college students version of the state−trait anxiety inventory. *Acta Sci. Nat. Univ. Pekin.* 31 108–114.

[B49] Liu P. Yang W. Yuan X. Bi C. Chen A. Huang X. (2015). Individual alerting efficiency modulates time perception. *Front. Psychol.* 6:386. 10.3389/fpsyg.2015.00386 25904881 PMC4387862

[B50] Martin B. (1961). The assessment of anxiety by physiological behavioral measures. *Psychol. Bull.* 58 234–255. 10.1037/h0045492 13767312

[B51] Matthews K. A. Manuck S. B. Saab P. G. (1986). Cardiovascular responses of adolescents during a naturally occurring stressor and their behavioral and psychophysiological predictors. *Psychophysiology* 23 198–209. 10.1111/j.1469-8986.1986.tb00618.x 3704076

[B52] Matthews W. J. Meck W. H. (2014). Time perception: the bad news and the good. *Wiley Interdiscip. Rev. Cogn. Sci.* 5 429–446. 10.1002/wcs.1298 25210578 PMC4142010

[B53] Mauss I. B. Bunge S. A. Gross J. J. (2007a). Automatic emotion regulation. *Soc. Pers. Psychol. Compass* 1 146–167.

[B54] Mauss I. B. Cook C. L. Gross J. J. (2007b). Automatic emotion regulation during anger provocation. *J. Exp. Soc. Psychol.* 43 698–711. 10.1016/j.jesp.2006.07.003

[B55] Mauss I. B. Wilhelm F. H. Gross J. J. (2004). Is there less to social anxiety than meets the eye? Emotion experience, expression, and bodily responding. *Cogn. Emot.* 18 631–642. 10.1080/02699930341000112

[B56] McLeod D. R. Hoehn-Saric R. Stefan R. L. (1986). Somatic symptoms of anxiety: comparison of self-report and physiological measures. *Biol. Psychiatry* 21 301–310. 10.1016/0006-3223(86)90051-X 3947711

[B57] Mella N. Conty L. Pouthas V. (2011). The role of physiological arousal in time perception: psychophysiological evidence from an emotion regulation paradigm. *Brain Cogn.* 75 182–187. 10.1016/j.bandc.2010.11.012 21145643

[B58] Meng X. Yang J. Cai Ay Ding X. Liu W. Li H. (2015). The neural mechanisms underlying the aging-related enhancement of positive affects: electrophysiological evidences. *Front. Aging Neurosci.* 7:143. 10.3389/fnagi.2015.00143 PMC4527238 26300770

[B59] Murata A. Moser J. S. Kitayama S. (2013). Culture shapes electrocortical responses during emotion suppression. *Soc. Cogn. Affect. Neurosci.* 8 595–601. 10.1093/scan/nss036 22422803 PMC3682443

[B60] Paul S. Simon D. Kniesche R. Kathmann N. Endrass T. (2013). Timing effects of antecedent- and response-focused emotion regulation strategies. *Biol. Psychol.* 94 136–142. 10.1016/j.biopsycho.2013.05.019 23747981

[B61] Peng H. Wu J. Sun X. Guan Q. Luo Y. (2018). Trait anxiety predicts the response to acute psychological stress. *Acta Psychol. Sin.* 50 997–1006. 10.3724/sp.j.1041.2018.00997

[B62] Penney T. B. Gibbon J. Meck W. H. (2000). Differential effects of auditory and visual signals on clock speed and temporal memory. *J. Exp. Psychol. Hum. Percept. Perform.* 26 1770–1787. 10.1037/0096-1523.26.6.1770 11129373

[B63] Pergamin-Hight L. Naim R. Bakermans-Kranenburg M. J. van IJzendoorn M. H. Bar-Haim Y. (2015). Content specificity of attention bias to threat in anxiety disorders: a meta-analysis. *Clin. Psychol. Rev.* 35 10–18. 10.1016/j.cpr.2014.10.005 25462110

[B64] Rajamma R. K. Paswan A. K. Hossain M. M. (2009). Why do shoppers abandon shopping cart? Perceived waiting time, risk, and transaction inconvenience. *J. Prod. Brand Manag*. 18 188–197. 10.1108/10610420910957816

[B65] Roemer L. Borkovec T. D. (1994). Effects of suppressing thoughts about emotional material. *J. Abnorm. Psychol.* 103 467–474. 10.1037/0021-843X.103.3.467 7930046

[B66] Shalom J. G. Israeli H. Markovitzky O. Lipsitz J. D. (2015). Social anxiety and physiological arousal during computer mediated vs. face to face communication. *Comput. Human Behav.* 44 202–208. 10.1016/j.chb.2014.11.056

[B67] Spielberger C. D. Gonzalez-Reigosa F. Martinez-Urrutia A. Natalicio L. F. S. Natalicio D. S. (2017). The state-trait anxiety inventory. *Rev. Interam. Psicol. J. Psychol.* 5 145–158.

[B68] Tamm M. Uusberg A. Allik J. Kreegipuu K. (2014). Emotional modulation of attention affects time perception: evidence from event-related potentials. *Acta Psychol. (Amst).* 149 148–156. 10.1016/j.actpsy.2014.02.008 24656420

[B69] Tian Y. Li L. Yin H. Huang X. (2019). Gender differences in the effect of facial attractiveness on perception of time. *Front. Psychol*. 10:1292. 10.3389/fpsyg.2019.01292 31231284 PMC6558225

[B70] Tian Y. Liu P. Huang X. (2018). The role of emotion regulation in reducing emotional distortions of duration perception. *Front. Psychol.* 9:347. 10.3389/fpsyg.2018.00347 29599740 PMC5862850

[B71] Tipples J. (2008). Negative emotionality influences the effects of emotion on time perception. *Emotion* 8:127. 10.1037/1528-3542.8.1.127 18266523

[B72] Törnqvist E. Månsson A. Larsson E. M. Hallström I. (2006). Impact of extended written information on patient anxiety and image motion artifacts during magnetic resonance imaging. *Acta Radiol.* 47 474–480. 10.1080/02841850600690355 16796309

[B73] Uusberg A. Naar R. Tamm M. Kreegipuu K. Gross J. J. (2018). Bending time: the role of affective appraisal in time perception. *Emotion* 18 1174–1188. 10.1037/emo0000397 29504799

[B74] Van Riel A. C. R. Semeijn J. Ribbink D. Bomert-Peters Y. (2012). Waiting for service at the checkout: negative emotional responses, store image and overall satisfaction. *J. Serv. Manag.* 23 144–169. 10.1108/09564231211226097

[B75] Van Volkinburg H. Balsam P. (2014). Effects of emotional valence and arousal on time perception. *Timing Time Percept.* 2 360–378. 10.1163/22134468-00002034 27110491 PMC4838289

[B76] Wilhelm F. H. Kochar A. S. Roth W. T. Gross J. J. (2001). Social anxiety and response to touch: incongruence between self-evaluative and physiological reactions. *Biol. Psychol.* 58 181–202. 10.1016/S0301-0511(01)00113-2 11698114

[B77] Williams L. E. Bargh J. A. Nocera C. C. Gray J. R. (2009). The unconscious regulation of emotion: nonconscious reappraisal goals modulate emotional reactivity. *Emotion* 9:847. 10.1037/a0017745 20001127 PMC2796590

[B78] Wilson C. J. Barnes-Holmes Y. Barnes-Holmes D. (2014). The effect of emotion regulation strategies on physiological and self-report measures of anxiety during a stress-inducing academic task. *Int. J. Psychol. Psychol. Ther.* 14 1–15. 10.1109/IEMBS.2000.897887

[B79] Yang Q. Tang P. Gu R. Luo W. Luo Y. J. (2015). Implicit emotion regulation affects outcome evaluation. *Soc. Cogn. Affect. Neurosci.* 10 824–831. 10.1093/scan/nsu124 25332404 PMC4448026

[B80] Yiend J. Mathews A. (2001). Anxiety and attention to threatening pictures. *Q. J. Exp. Psychol. Sect. A* 54 665–681. 10.1080/713755991 11548029

[B81] Yin D. Liu W. Zeljic K. Lv Q. Wang Z. You M. (2017). Failure in cognitive suppression of negative affect in adolescents with generalized anxiety disorder. *Sci. Rep.* 7:6583. 10.1038/s41598-017-07063-5 28747683 PMC5529377

[B82] Yoo J.-Y. Lee J.-H. (2015). The effects of valence and arousal on time perception in individuals with social anxiety. *Front. Psychol.* 6:1208. 10.3389/fpsyg.2015.01208 26347679 PMC4538238

[B83] Yuan J. Ding N. Liu Y. Yang J. (2015a). Unconscious emotion regulation: nonconscious reappraisal decreases emotion-related physiological reactivity during frustration. *Cogn. Emot.* 29 1042–1053. 10.1080/02699931.2014.965663 25297822

[B84] Yuan J. Zhang J. Zhou X. Yang J. Meng X. Zhang Q. (2012). Neural mechanisms underlying the higher levels of subjective well-being in extraverts: pleasant bias and unpleasant resistance. *Cogn. Affect. Behav. Neurosci.* 12 175–192. 10.3758/s13415-011-0064-8 21987094

[B85] Yuan J. Long Q. S. Ding N. X. Lou Y. X. Liu Y. Y. Yang J. M. (2015b). Suppression dampens unpleasant emotion faster than reappraisal: neural dynamics in a Chinese sample. *Sci. China Life Sci.* 58 480–491. 10.1007/s11427-014-4739-6 25316046

[B86] Zakay D. Block R. A. (1996). The role of attention in time estimation processes. *Adv. Psychol.* 115 143–164. 10.1016/s0166-4115(96)80057-4

